# Gadolinium in pediatric cardiovascular magnetic resonance: what we know and how we practice

**DOI:** 10.1186/1532-429X-14-56

**Published:** 2012-08-07

**Authors:** Howard Meng, Lars Grosse-Wortmann

**Affiliations:** 1 The Labatt Family Heart Centre, The Hospital for Sick Children, 555 University Avenue, Toronto, ON, M5G 1X8, Canada

## Abstract

**Background:**

The association of gadolinium-based contrast agents (GBCAs) with nephrogenic systemic fibrosis (NSF) has led to a heightened awareness towards patients’ renal function. Whereas detailed guidelines exist for the use of GBCAs in adult patients, best practice is less well defined in children, especially in the very young. We aimed at identifying current practice with regards to the use of GBCAs in children who undergo Cardiovascular Magnetic Resonance.

**Methods:**

We conducted a worldwide survey among cardiac imagers with pediatric expertise. The questionnaire contained 21 questions covering the imagers’ work environments, GBCAs used, monitoring of renal function, and a special emphasis was placed on the practice in neonates.

**Results:**

The survey yielded 70 replies. The single most commonly used GBCA was gadopentetate dimeglumine 34/70 (49%). Among the respondents, the choice of GBCA was more importantly based on availability 26/70 (37%) and approval by a pharmaceutical licensing body that most closely reflects the indication 16/70 (23%) than image quality 7/70 (10%) and side effect profile 8/70 (11%). 55/70 (79%) of respondents performed scans in neonates <1 week of age and 52/55 (95%) of them used GBCA in neonates. 65/70 (93%) respondents at least assess some of their patients’ renal functions. Formula-based estimate of glomerular filtration rate is the most popular assessment method 35/65 (54%). In patients with a glomerular filtration rate < 30 ml/min/1.73 m^2^ 62/70 (89%) of respondents do not administer gadolinium at all. The single most common side effect of gadolinium was noted to be nausea/emesis 34/57 (60%) followed by discomfort at injection site 17/57 (30%).

**Conclusions:**

Cardiac imagers are aware of the immature renal function and physiological differences of their pediatric patients that place them at risk for NSF. Epidemiological data is needed for pediatric cardiovascular licensure of gadolinium compounds and for the creation of practice guidelines which will replace current-day practice based on individual clinical judgment.

## Background

For the majority of Cardiac Magnetic Resonance (CMR) indications, the application of gadolinium is required. Over the years, numerous gadolinium-based contrast agents (GBCAs) have been introduced. Although commonly considered safe in the majority of patients, reports of nephrogenic systemic fibrosis (NSF) since 2000 have led to a heightened awareness towards the adverse effects of GBCAs. Explicit licensing for cardiac indications is not available for gadolinium compounds in adults, let alone in children, although in the United Kingdom, Magnevist is indicated for whole body applications which can be interpreted to include the heart. In addition, no GBCA has been approved by the Food and Drug Administration (FDA) for use in children <2 years of age, including non-cardiac indications. [1] As a consequence, radiologists and cardiologists practice contrast–based CMR, particularly in young children, using their best judgment of the risk of adverse reactions *versus* the benefit of the investigation for the patient’s management. We aimed at evaluating the current practice involving the use of GBCAs among pediatric cardiac imagers. Specifically, we were interested in finding out which GBCAs are most commonly used and what type of precautions are undertaken to avoid adverse drug reactions.

## Methods

We conducted a survey among pediatric imaging specialists (cardiologists and radiologists) around the world. These individuals were identified from three sources: The directories of the pediatric working group of the Society of Cardiovascular Magnetic Resonance and of the imaging working group of the Association for European Pediatric Cardiology, as well as from personal contacts. See Additional file 1: Gadolinium Survey for the questionnaire. The survey contained 21 questions covering the imagers’ work environments, GBCAs used, and monitoring of renal function. Special emphasis was placed on practice in neonates. In total, 175 specialists were emailed a link to a web-based survey. Of the 175 email recipients, 22 emails were rendered “undeliverable” by our emailing system likely due to incorrect or out dated email addresses. The survey was open for responses for one month. As practices may vary among imagers within the same institution, more than one imager was contacted at select centers.

## Results

**Figure 1:**
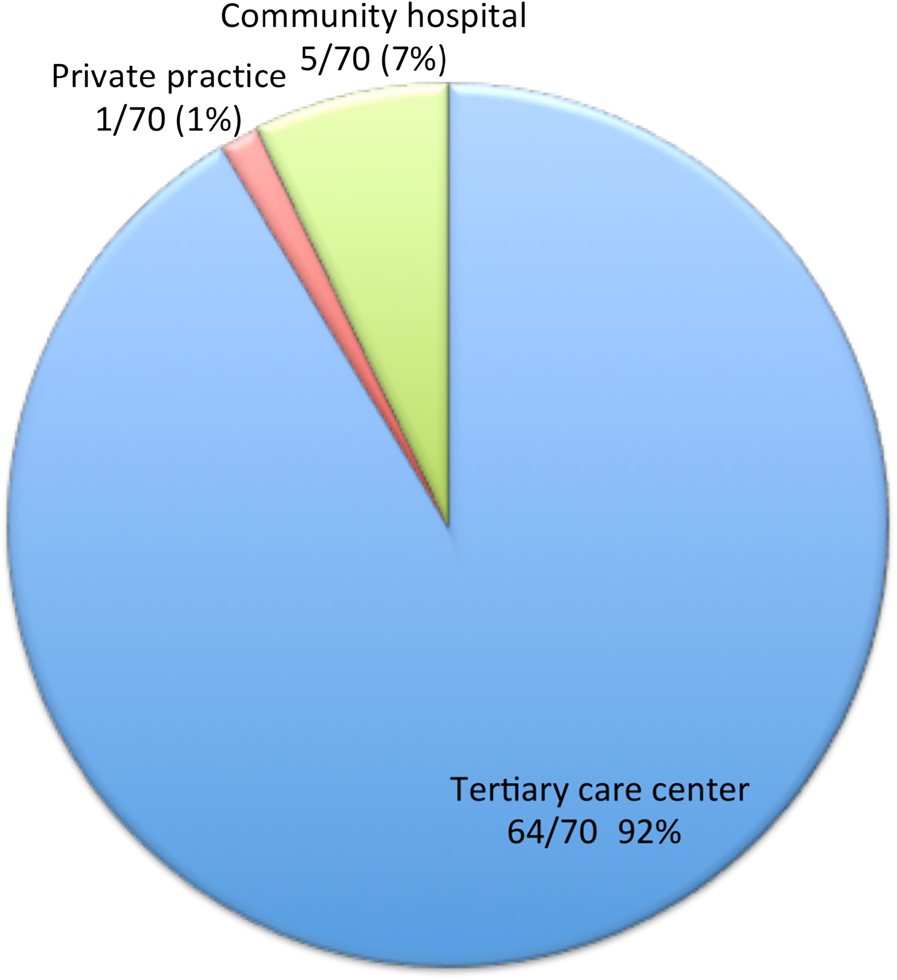
** Practice setting of respondents.** The majority 92% practice in “tertiary care centers”, with 7% practicing in “community hospitals” and 1% in “private practice”.

**Figure 2:**
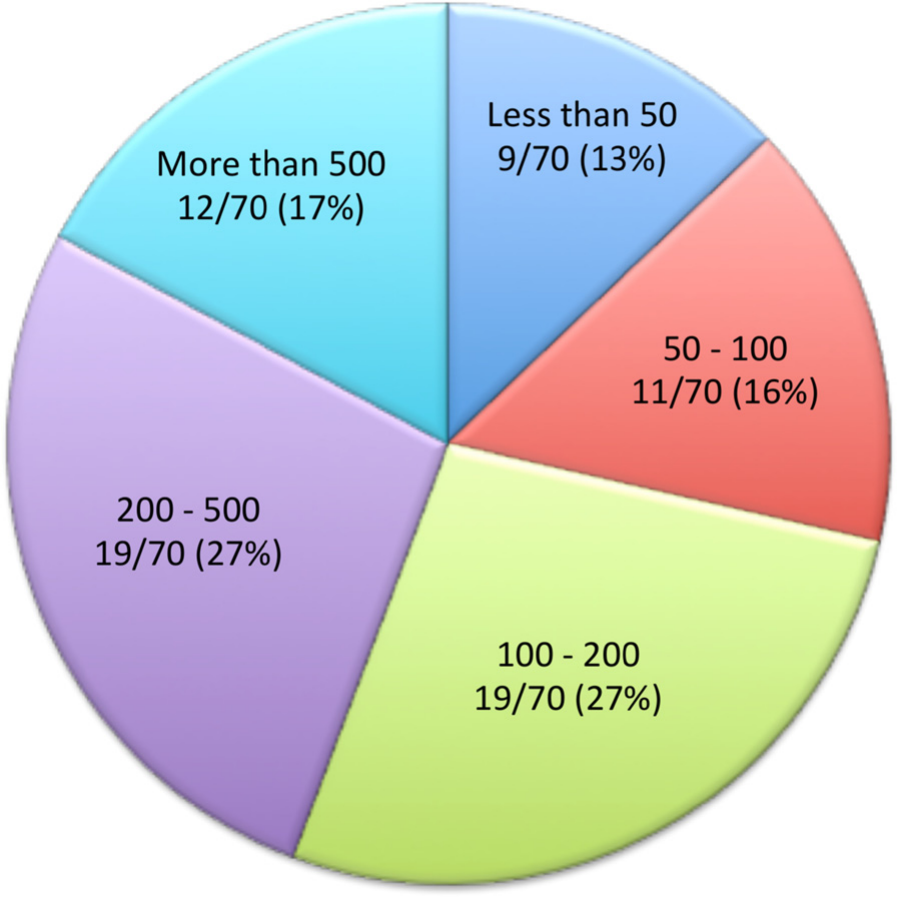
** Number of pediatric CMRs performed each year.** Respondents who performed “100-200” and “200-500” pediatric CMRs each made up 27% of all respondents. This is followed by “more than 500” (17%), “50-100” (16%), and “less than 50” (13%).

**Figure 3:**
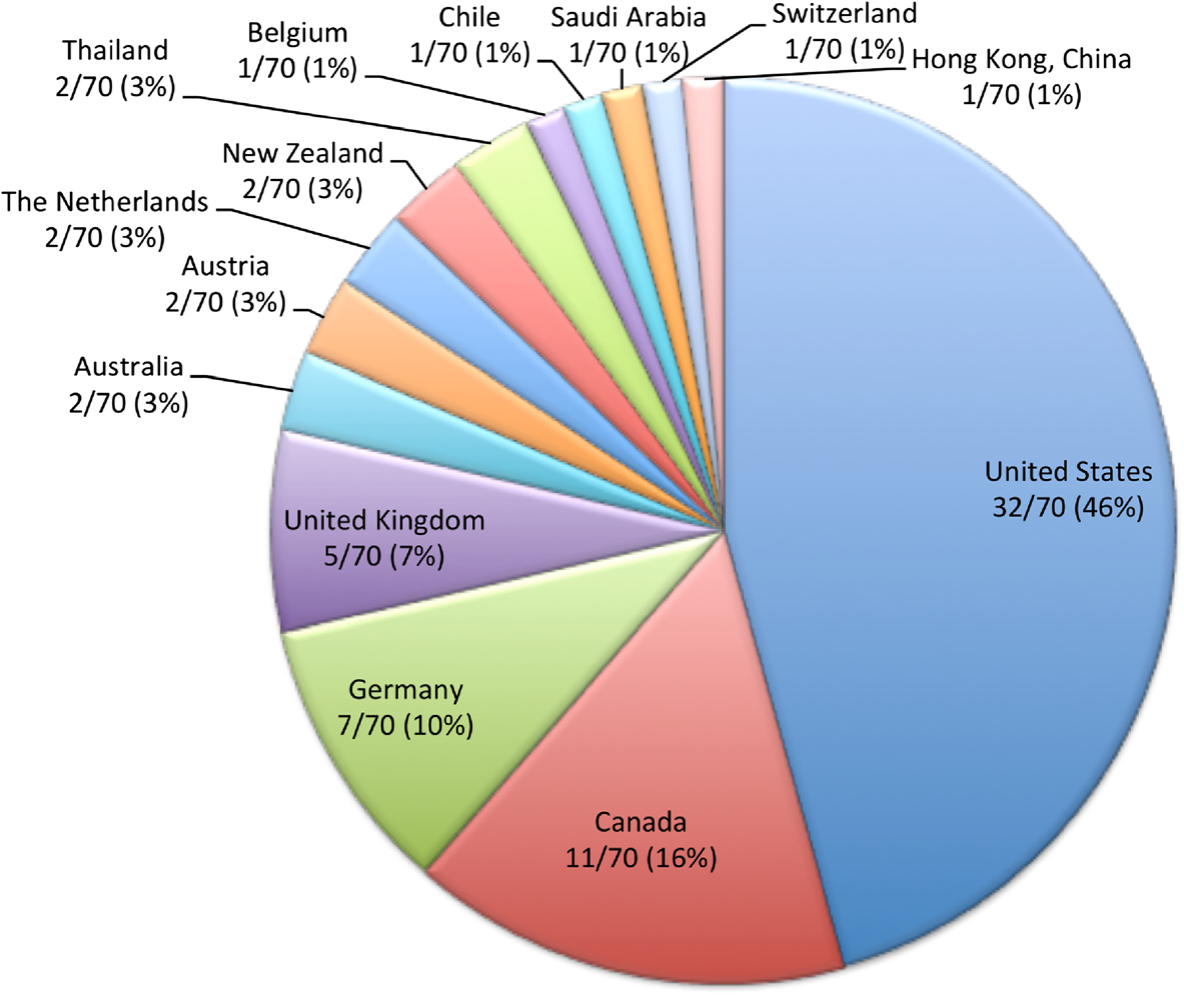
** Respondents’ country of origin.** The majority of respondents are from North America: United States (46%) and Canada (16%).

Our survey produced 70 responses for a response rate of 70/153 (46%). Of the 70 respondents, 26/70 (37%) were radiologists and 44/70 (63%) were cardiologists. The work settings of respondents are displayed in Figure 1, as well as the annual number of pediatric CMR studies performed, Figure 2. Responses were received from around the world with the majority of respondents from North America and Europe (Figure 3).

**Figure 4:**
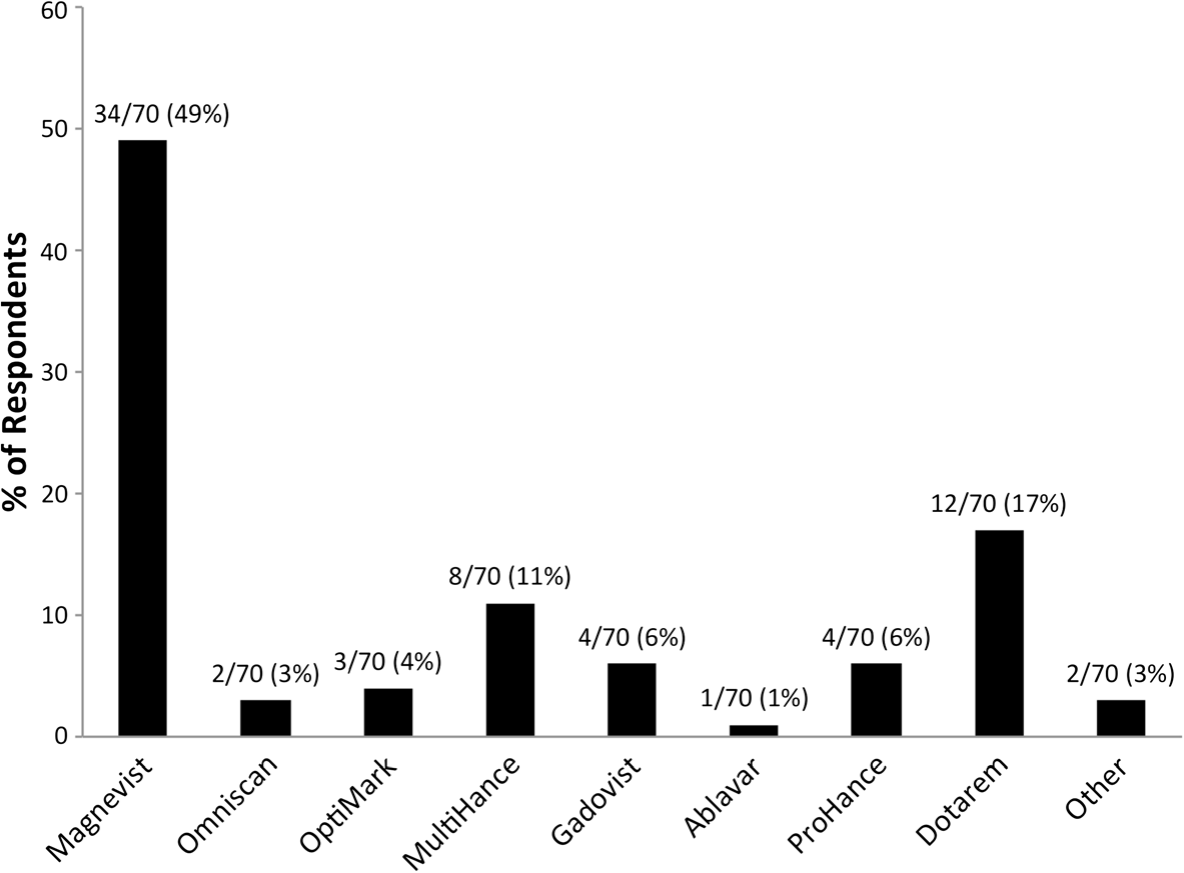
** Most commonly used GBCA.** “Magnevist” (49%), “Dotarem” (17%), and “MultiHance” (11%) are the three most commonly used.

**Figure 5:**
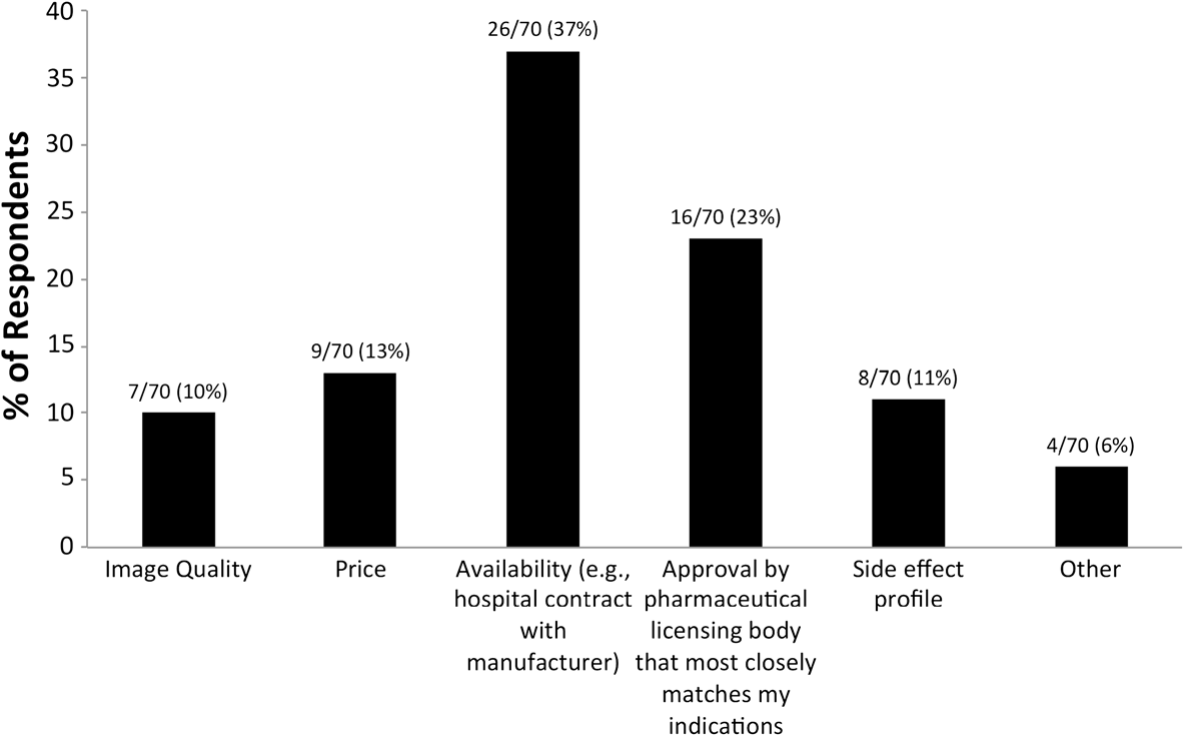
** Rationale for use of particular GBCA.** 37% of respondents indicated “availability (*e.g.* hospital contract with manufacturer)” as the most important reason, followed by “approval by pharmaceutical licensing body that most closely matches my indications” (23%). Less important reasons for particular GBCA use are “price” (13%), “side effect profile” (11%), “image quality” (10%), and “other” (6%).

For easier reference we will refer to the individual GBCAs under their brand name in the remainder of the text. The single most commonly used GBCAs were Magnevist 34/70 (49%), followed by Dotarem 12/70 (17%) and MultiHance 8/70 (11%) (Figure 4). Rationales for using specific GBCAs are given in Figure 5. 26/70 (37%) of respondents chose “availability of the product” as the single most important reason for using a particular GBCA, followed by “approval by pharmaceutical licensing body that most closely matches my indications” at 16/70 (23%).

55/70 (79%) of the respondents performed scans in neonates <1 week of age and 52/55 (95%) of them use GBCA in these patients. Commonly used GBCAs in neonates were Magnevist 23/48 (48%), Dotarem 10/48 (21%), and MultiHance 8/48 (17%). 42/51 (82%) of GBCA users in neonates used the same brand of GBCA as for older children and adolescents.

With NSF as the most severe GBCA related adverse effect with the exception of anaphylactic reactions, we assessed the respondents’ practice around the protection of kidney function. To determine renal function, the primary method of assessment was a formula-based estimate of glomerular filtration rate (GFR) (35/65 or 54%). Serum creatinine concentration was used by 20/65 (31%) of respondents and quantification of urine output by 4/65 (6%). Other methods of assessment included the use of Cystatin C by one respondent and a combination of data, including renal ultrasound if available (1/65) 65/70 (93%) evaluated renal function in some or all of their patients. Where renal function assessments were performed selectively (49/70 of 70%), these were done in patients with active renal disease and/or impaired renal function 47/49 (96%), patients with a risk factor for impaired renal function (*e.g.* vasculitis, arteriopathies) but no known renal dysfunction 39/49 (79%), and patients with impaired renal function in the past 39/49, (79%). “All neonates” and “all patients <1 week of age” were evaluated for renal function by 16/49 (33%) and 15/49 (31%), respectively. In patients with severe renal failure (GFR <30 ml/min/1.73 m^2^), 61/70 (87%) of respondents chose not to administer gadolinium at all. A specific GBCA was used by 4/70 (6%) of respondents. 2/70 (3%) gave gadolinium, but never double dose. No respondents claimed to maintain the same practice in patients with a GFR <30 ml/min/1.73 m^2^ as in patients with normal or near-normal renal function.

**Figure 6:**
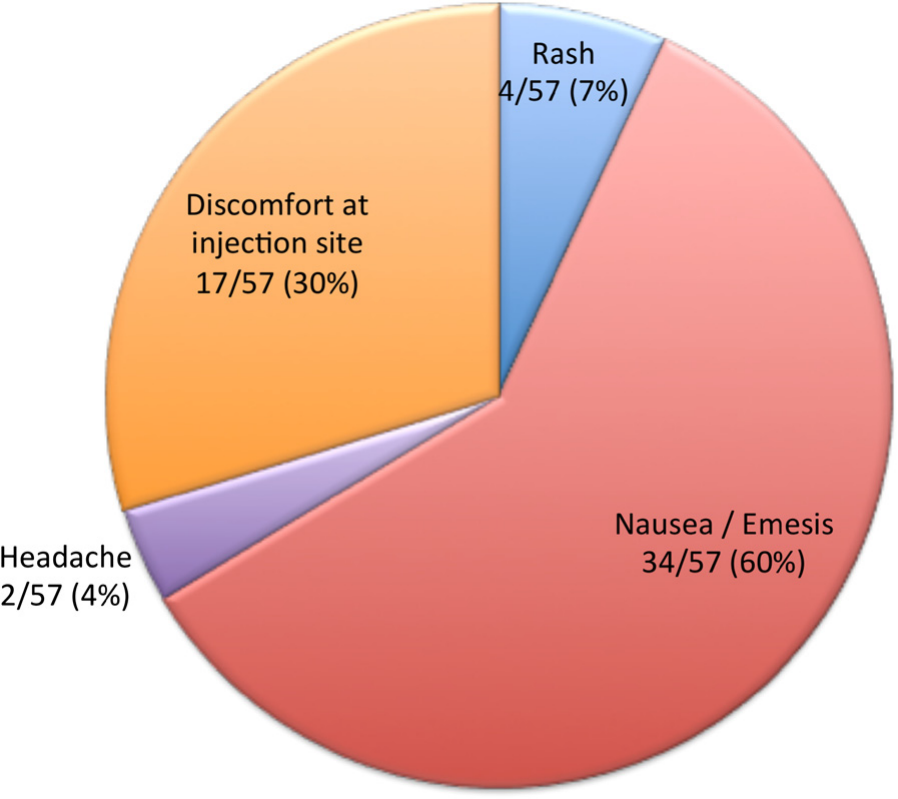
** Most common side effect of GBCA use.** “Nausea/emesis” (60%) and “discomfort at injection site” (30%) are reported most frequently. “Rash” (7%), “headache” (4%), and “light-headedness” (0%) are experienced rarely, if at all.

In patients with a GFR between 30 and 60 ml/min 1.73 m^2^, 14/70 (20%) of respondents refrained from giving any GBCA at all while 8/70 (11%) of respondents identified their practice is unchanged as compared to a GFR >60 ml/min 1.73 m^2^. 22/70 (31%) of respondents indicated the use of GBCA but never “double dose”. In cases with renal insufficiency, MultiHance 8/25 (32%), Dotarem 7/25 (28%), and Magnevist 5/25 (20%) were the most commonly used specific agents. In contrast, Omniscan, OptiMark, and Ablavar were not used by any of our respondents in patients with renal failure. 29/67 (43%) of respondents never obtained written consent for GBCA application while 14/67 (21%) did on occasion and 23/67 (34%) always (1/67 - 1% obtained written consent “most of the time”). 12/69 (17%) of respondents claimed they have never encountered a patient with a GBCA-attributed side effect and 57/69 (83%) rarely (1-5% of cases) encountered one. The two most common side effects from GBCA administration in the respondents’ practice were nausea/emesis 34/57 (60%) and discomfort at injection site 17/57 (30%). Rash 4/57 (7%), headache 2/57 (4%), light-headedness 0/57 (0%), and bronchospasm 0/57 (0%) were much less frequent (Figure 6).

## Discussion

Our survey revealed that three different formulas of GBCAs make up the bulk of contrast media used for pediatric CMR worldwide today: Magnevist, Dotarem, and MultiHance. Immediate concerns around patient safety may not be the decisive factor in selecting specific GCBA for use. When asked about the single most important reason for using a particular agent, availability of the product featured most prominently (37% of respondents). Interestingly, image quality and side effect profile were less (11% each) determinant of the type of GBCA used. We speculate that the side effect profile is of lesser significance when choosing an agent because of the relatively low overall incidence of adverse reactions. A single center retrospective study identified adverse drug reactions (ADR) to be 0.0404% based on 158,439 doses administered [2]. It should be remembered, however, that in subpopulations, such as the very young, patients with renal and/or hepatic impairment, and cardiovascular diseases, GBCAs carry much higher ADR rates [3]. Voth and Hahn and their respective colleagues both reported side effects in as many as 5.8% of all children, more than ten times that of adults [3,4] and comparable to a neuro-imaging based study by Lowe *et al.*[5]. Conversely, a multicenter study of 432 children between 6 months to 18 years of age reported only 1 mild event after Omniscan or Magnevist application at 0.1 mmol/kg [6]. In our survey, and consistent with previous reports [7], most respondents identified rare (1-5%) or no side effects associated with GBCA injection. The majority of these side effects are mild, mainly consisting of nausea/emesis 34/57 (60%) and discomfort at the injection site 17/57 (30%).

Magnevist was the primary agent in the practice of approximately half of the cardiac imagers in this survey, while Omniscan was the first line agent of only 2/70 (3%) of respondents. Omniscan, which held an overall market share of 34% in 2006, fell out of favor with the establishment of an association with NSF [8]. First reported in 2000, this systemic condition is characterized by a fibrosing dermatopathy in patients with kidney failure. The discovery of fibrosis in joints, liver, and other end organs resulted in a name change to nephrogenic systemic fibrosis [9]. The effects of NSF are debilitating and many patients become wheelchair bound. In 2006, Grobner *et al.* suggested a link between GBCAs, renal failure, and NSF [10].

It is free gadolinium that has classically been associated with the occurrence of NSF [8]. Therefore, the more stable the chelate-gadolinium compound the safer the agent is assumed to be with regards to NSF. Linear chelates are considered to have inferior structural stability when compared to GBCAs with a cyclic chelate structure [11]. Unconfounded cases of NSF have been identified with Omniscan, Magnevist, and Optimark, all of which are linear. Conversely, cyclic compounds have been associated with few, if any cases of NSF [8]. In addition to the molecular configuration of the chelate, ionic chelates, such as Magnevist and MultiHance provide greater stability than non-ionic chelates, including Optimark and Omniscan. Recently, it has been suggested that not only free, but also chelated GBCA can trigger a pro-inflammatory and pro-fibrotic cascade [12] so that factors other than the chelate structure likely play a role in the pathogenesis of NSF.

Given their less-well-developed blood–brain barrier and immature renal function, the use of GBCA in neonates has been cautioned [6]. According to the most recent FDA information, Omniscan, Magnevist, MultiHance, Gadovist, and Prohance have been approved for use in pediatric patients >2 years of age, while no GBCA has been approved for use in children <2 years. Similarly, the European Medicines Agency (EMA) has classified the use of ‘high-risk’ GBCAs in neonates less than 4 weeks of age as contraindicated and cautioned the use of any GBCA in pediatric patients less than 1 year of age [13]. Although no agent was specifically licensed for the use in this patient population, the Commission on Human Medicines and Pharmcovigilance Expert Advisory Group, both of which are part of the Medicines and Healthcare products Regulatory Agency in the UK advocated for the use of lowest possible dose of medium-risk (MultiHance, Primovist, Ablavar) or low-risk agents (Gadovist, ProHance, Dotarem) for neonates in 2010. A large portion of the respondents to our survey (79%) uses GBCAs in neonates. Interestingly, we observed only minor differences between the single most preferred agents in neonates *versus* those used in older children. The biggest differences between the two patient populations were seen for Dotarem and MultiHance, which both increased in “popularity” from 17% to 21% and 11% to 19%, respectively, with the use in neonates. Dotarem, as an ionic cyclic chelate, is theorized to provide better molecular stability which may be of benefit in patients who are potentially more susceptible to NSF. According to suggestions by Riccabona *et al.* for GBCA use in infants, only cyclic compounds should be used [14]. It is more difficult to explain the increased use in MultiHance as it is based on a linear (although ionic) chelate and has not been approved for use in patients <18 years of age.

The discovery of kidney failure as a major contributing factor to the development of NSF has heightened awareness towards renal function before gadolinium administration [15,16]. Gadolinium is primarily excreted by the kidneys (>95%). Under normal renal function, the half-life of most GBCAs is approximately 1.6 hours, with >95% of gadolinium cleared from the body in 24 hours [17]. The notable exception among the commonly used agents is Ablavar with a half-life of 15 hours [18]. In patients with moderate and severe renal insufficiency the half-life of GBCA averages 5.6 and 9.2 hours, respectively [19], allowing more time for the generation of and exposure to free gadolinium.

In general, healthy adults have a normal GFR ≥ 120 ml/min/1.73 m^2^. According to ACR, CAR, and EMEA GBCAs should be avoided in patients with acute renal dysfunction and/or a GFR < 30 ml/min/1.73 m^2^, if possible. GBCA is only to be used when it is vitally important for the patient’s health. The lowest dose possible for imaging must be given and GBCAs classified as “high risk” or “group I” by the EMA and ACR, respectively, including Magnevist, Optimark and Omniscan, are not recommended.

At Massachusetts General Hospital, and mirroring another group’s results, no new patients were identified with NSF following implementation of renal function screening [16,20].

93% of pediatric cardiac imagers who completed the survey estimate GFR in some or all of their patients. Those who use GFR estimates selectively are primarily concerned about patients with impaired renal function at present (96%) or in the past (79%), as well as about patients with a risk factor for impaired renal function (79%). In comparison, very young age alone was seen as a reason to check GFR among a much lower proportion of respondents.

The ACR recommends applying the adult guidelines to pediatric patients [1]. In light of the physiologically low GFR in neonates, this would prevent most of these patients from undergoing CMR. The average GFR in full term babies is 26 ml/min 1.73 m^2^, which is similar to chronic kidney disease class 4 (<30 ml/min 1.73 m^2^). Neonates with congenital heart disease who need cardiac imaging very early in life often struggle with problems of prematurity, low cardiac output, as well as electrolyte and/or acid–base disturbances, all which may result in an even lower GFR. With this in mind, it is interesting to note that most imaging experts would not give gadolinium to an older child or adult with similar GFR, but do so frequently in neonates (at least when renal function is not assessed). Should we be concerned about giving gadolinium to neonates or do they not share the same NSF risk of older individuals with similarly low GFRs because their condition is physiologic? Also, is the Schwartz formula the adequate tool for assessing neonatal renal function? Many factors make accurate renal function assessment in neonates challenging: Perinatal adaptation is a highly individualized and dynamic process, confounding any comparison with normal values. Immediately after birth, the newborn’s serum creatinine concentration mirrors the maternal creatinine levels, rather than indicating neonatal renal function [21].

54% of respondents indicated the Schwartz formula to be their primary method of renal function evaluation in neonates, although the shortcomings of this method in neonates are well recognized. Hahn *et al.* argued that, while excretion rates are lower in newborns as compared to adults, more gadolinium per body weight is eliminated, possibly reducing the risk of NSF despite a low estimated GFR. In 2009, the Canadian Association of Radiologists stated that “an estimation of GFR using serum creatinine levels in children and neonates is not optimal”, recommended to use “regional judgement” and recognized the need for refined guidelines in the future [22]. As an alternative to the Schwartz formula, 31% of respondents indicated the use of serum creatinine and 6% indicated urine output. Cystatin C-based estimates promise to be a better estimate of GFR in children than the Schwartz formula [23], but pediatric reference values for Cystatin C have not yet been established [24].

## Conclusions

Expert cardiac imagers are aware of the risk of NSF in their pediatric patients. At the same time, practice heterogeneity around the use of NSF in newborn patients is prevalent. Epidemiological data is needed for pediatric cardiovascular licensure of gadolinium compounds and for the creation of practice guidelines which will replace current-day practice based on individual clinical judgement. Currently, a trial with Gadovist including patients less than 2 years of age is underway. In the meantime, a balanced discussion of the use of GBCA in neonates must take into account the diagnostic value of CMR in the management of very young patients with congenital heart disease, such as obstructive lesions of the aortic arch, and pulmonary atresia with major pulmonary collateral arteries.

## Competing interests

The authors declare that they have no competing interest.

## Authors’ contributions

HM conducted the survey, analyzed the data, and drafted the manuscript. LGW conceived and designed the study, as well as revised the manuscript. Both authors read and approved the manuscript.

## Supplementary Material

Additional file 1 Gadolinium Survey – questionnaire. Click here for file
