# Supplementary material for: Gadolinium in pediatric cardiovascular magnetic resonance: what we know and how we practice
Source: J Cardiovasc Magn Reson. 2012 Aug 7;14(1):56. doi: 10.1186/1532-429X-14-56 (PMC3492017; doi:10.1186/1532-429X-14-56)
Supplement: Additional file 1 — Gadolinium Survey – questionnaire. [file 1532-429X-14-56-S1.pdf]

Gadolinium Survey

* Required

1. Are you a radiologist or cardiologist ? *

- Radiologist
- Cardiologist

2. How many pediatric cardiac MRI studies do you perform in a year ? *

- Less than 50
- 50 - 100
- 100 - 200
- 200 - 500
- More than 500

3. What setting do you work in ? *

- Tertiary care center
- Community hospital
- Private practice

4. Which country do you work in? *

5. Which gadolinium agent(s) do you use in clinical practice (check all that apply) ? *

- Gadopentetatedimeglumine (Magnevist)
- Gadodiamide (Omniscan)
- Gadoversetamide (OptiMARK)
- Gadobenatedimeglumine (MultiHance)
- Gadobutrol (Gadovist)
- Gadofosveset (Ablavar)
- Gadoteridol (ProHance)
- Gadoterate (Dotarem)
- Other:

6. Which single contrast agent do you most commonly use (check one) ? *

- Gadopentetatedimeglumine (Magnevist)
- Gadodiamide (Omniscan)
- Gadoversetamide (OptiMARK)
- Gadobenatedimeglumine (MultiHance)
- Gadobutrol (Gadovist)
- Gadofosveset (Ablavar)
- Gadoteridol (ProHance)
- Gadoterate (Dotarem)
- Other:

7. What are your reasons for using this particular agent (question 6) over others in your practice (check all that apply) ? *

- Image quality
- Price
- Availability
- Approval by pharmaceutical licensing body that most closely matches my indications
- Side effect profile
- Other:

8. What is the single most important reason for using this particular agent over others in your practice (check only one) ? *

- Image quality
- Price
- Availability (e.g., hospital contract with manufacturer)
- Approval by pharmaceutical licensing body that most closely matches my indications
- Side effect profile
- Other:

9. Do you perform scans in neonates < 1 week ? (If no, please go to question 13) *

- Yes
- No

10. If you answered "yes" to question 9, do you use gadolinium in neonates < 1 week ? (If no, please go to question 13)

- Yes
- No

11. If you answered "yes" to question 10, which gadolinium agent(s) do you use in neonates < 1 week ?

- Gadopentetatedimeglumine (Magnevist)
- Gadodiamide (Omniscan)
- Gadoversetamide (OptiMARK)
- Gadobenatedimeglumine (MultiHance)
- Gadobutrol (Gadovist)
- Gadofosveset (Ablavar)
- Gadoteridol (ProHance)
- Gadoterate (Dotarem)
- Other:

12. Does / do this / these gadolinium agent(s) differ from one(s) used in older children and adolescents ?

- Yes
- No

13. Which single method do you primarily use to assess renal function in neonates < 1 week ? *

- Serum creatinine
- Glomerular filtration rate (GFR, Schwartz formula)
- Urine output
- Other:

14. Do you estimate GFR (based on serum creatinine) in none, some or all of your patients ? *

- None
- Some
- All

15. If your answer to the previous question was “some”, in whom do you obtain a creatinine to calculate GFR ?(check all that apply)

- ☐ All neonates
- ☐ All patients < 1 week of age
- ☐ All patients with active renal disease and / or impaired renal function
- ☐ Patients with impaired renal function in the past
- ☐ Patients with a risk factor for impaired renal function (vasculitis, arteriopathies), but no known renal dysfunction

16. Please check all that apply: In patients with GFR <30ml/kg/1.73m^2, *

- ☐ I give gadolinium, but use a specific agent (Please indicate agent in question 18)
- ☐ I give gadolinium, but never 'double dose'
- ☐ I do not administer gadolinium at all
- ☐ My practice is unchanged as comapred to a GFR >60ml/kg/1.73m^2
- ☐ Other: ☐

17. Please check all that apply: In patients with GFR between 30 and 60ml/kg/1.73m^2, *

- ☐ I give gadolinium, but use a specific agent (Please indicate agent in question 18)
- ☐ I give gadolinium, but never “double dose”
- ☐ I do not administer gadolinium at all
- ☐ My practice is unchanged as compared to a GFR > 60ml/kg/1.73m^2
- ☐ Other: ☐

18. If you selected "I give gadolinium, but use a specific agent" for questions 16 or 17, please indicate the agent used

- ○ Gadopentetatedimeglumine (Magnevist)
- ○ Gadodiamide (Omniscan)
- ○ Gadoversetamide (OptiMARK)
- ○ Gadobenatedimeglumine (MultiHance)
- ○ Gadobutrol (Gadovist)
- ○ Gadofosveset (Ablavar)
- ○ Gadoteridol (ProHance)

- Gadoterate (Dotarem)
- Other:

19. Do you obtain written consent before administering gadolinium? *

- Never
- Sometimes
- Most of the time
- Always

20. What is the single most common side effect of gadolinium in your experience *

- Rash
- Nausea / Emesis
- Light-headedness
- Headache
- Bronchospasm
- Discomfort at injection site

21. How frequently do you encounter one or more side effects attributable to gadolinium ? *

- Never
- Rarely (1-5% of cases)
- Sometimes (5-20% of cases)
- Frequently (>20% of cases)

Comments [Optional]

Submit
